# Treatment of Urethral Strictures from Irradiation and Other Nonsurgical Forms of Pelvic Cancer Treatment

**DOI:** 10.1155/2015/476390

**Published:** 2015-10-07

**Authors:** Iyad Khourdaji, Jacob Parke, Avinash Chennamsetty, Frank Burks

**Affiliations:** ^1^Beaumont Health System, Department of Urology, 3601 W. Thirteen Mile Road, Royal Oak, MI 48073, USA; ^2^Oakland University William Beaumont School of Medicine, 216 O'Dowd Hall, 2200 N. Squirrel Road, Rochester, MI 48309, USA

## Abstract

Radiation therapy (RT), external beam radiation therapy (EBRT), brachytherapy (BT), photon beam therapy (PBT), high intensity focused ultrasound (HIFU), and cryotherapy are noninvasive treatment options for pelvic malignancies and prostate cancer. Though effective in treating cancer, urethral stricture disease is an underrecognized and poorly reported sequela of these treatment modalities. Studies estimate the incidence of stricture from BT to be 1.8%, EBRT 1.7%, combined EBRT and BT 5.2%, and cryotherapy 2.5%. Radiation effects on the genitourinary system can manifest early or months to years after treatment with the onus being on the clinician to investigate and rule-out stricture disease as an underlying etiology for lower urinary tract symptoms. Obliterative endarteritis resulting in ischemia and fibrosis of the irradiated tissue complicates treatment strategies, which include urethral dilation, direct-vision internal urethrotomy (DVIU), urethral stents, and urethroplasty. Failure rates for dilation and DVIU are exceedingly high with several studies indicating that urethroplasty is the most definitive and durable treatment modality for patients with radiation-induced stricture disease. However, a detailed discussion should be offered regarding development or worsening of incontinence after treatment with urethroplasty. Further studies are required to assess the nature and treatment of cryotherapy and HIFU-induced strictures.

## 1. Introduction

Radiation therapy (RT) is a well-known and effective means of treating pelvic malignancies. External beam radiation therapy (EBRT), brachytherapy (BT), photon beam therapy (PBT), high intensity focused ultrasound (HIFU), and cryotherapy are forms of noninvasive treatments for malignancy. Although an effective form of cancer treatment, radiation therapy is not without complication. Urethral stricture disease is an underrecognized and poorly reported complication that can cause severe morbidity for cancer survivors [1, 2]. Radiated urethral tissue in particular poses a challenge for the reconstructive urologist. It is our goal to provide a comprehensive discussion of etiology, incidence, and available treatment options for urethral stricture disease following pelvic radiation.

## 2. Epidemiology

The term stricture has previously been the nomenclature applied to any narrowing along the entirety of the urethra. Updated terminology now uses* stenosis* and* stricture* to more appropriately localize the abnormality along the urethra. Narrowed segments of the urethra surrounded by spongiofibrosis have been deemed* stricture*. In contrast, constrictions that occur within the posterior urethra are deemed* stenosis* [3]. It is import to differentiate between the two as treatments can differ depending on location [4].

Radiation effects on the genitourinary system can manifest early after treatment or present months or years after. Acutely, radiation treatment can cause lower urinary tract symptoms (LUTS) such as frequency, urgency, and dysuria requiring symptomatic management [5, 6]. Late urinary toxicity is a prolonged sequelae that can present with hesitancy, retention, stricture, and hematuria [5]. The timing of late toxicity is highly varied and can declare itself decades after initial radiation treatment [7]. Although narrowing can theoretically form at any location along the course of the urethra, bulbomembranous stenosis accounts for 90% of reported strictures after RT [8]. A study investigating the CaPSure registry reported the incidence of stricture from four separate categories of treatment. Their study found the incidence of stricture from BT to be 1.8%, EBRT 1.7%, combined EBRT and BT 5.2%, and cryotherapy as 2.5% [9]. A more recent study documented ranges of bulbomembranous stricture incidence from BT at 1 to 8% versus 2 to 4% for EBRT [2]. Data reporting the incidence of cryotherapy-related stricture disease is also limited; however a recent study comparing 10-year propensity-weighted adverse urinary events after treatment for prostate cancer found incidence of stricture to be 1.05% (*n* = 2115) [10].

## 3. Etiology

Radiation therapy causes damage on living cells in two main ways: directly, inflicting damage to cellular DNA initiating DNA mutation and apoptosis, and indirectly, interacting with free water within the cell to form hydroxyl free radicals that are highly unstable within the cell. Furthermore, cells that are actively dividing are more sensitive to ionizing radiation than those more stagnant in the cell cycle [11]. Data reviewing the pathophysiology behind urethral stricture in inflammatory, autoimmune, and infectious processes is well-understood and well-described [8]. Unfortunately, studies investigating the underlying mechanism causing stricture after radiation therapy are limited.

Ballek and Gonzalez have studied and described the pathophysiology pertaining to radiation-induced strictures in great detail. Through the aforementioned mechanisms, basement membranes of vascular tissues supplying the urethra become damaged, resulting in occlusion, thrombosis, and impaired neovascularization. Vascular compromise leads to inadequate tissue perfusion and poor wound healing. The result is fibroblasts that are rendered incapable of producing collagen to meet the demands of the healing wound. Collagen maturation is also compromised by poorly functioning fibroblasts leading to contraction and scar formation [12]. Studies have demonstrated this effect to be long lasting and even transmitted to daughter fibroblasts within tissue [13]. Over time, the corpus spongiosum is replaced with fibrotic tissue and subsequent occlusion of the urethral lumen occurs [12].

Healing of these compromised tissues should also be a consideration of the urologist when considering surgical intervention such as reconstructive urethroplasty. Patients receiving radiation therapy weeks to months prior to undergoing surgical intervention experience poor wound healing compared to those who receive similar doses of radiation 6 months or more before surgery [13, 14]. Gorodetsky further found this effect to be dose-dependent; as radiation dose was increased, wound strength decreased. Tissue planes can be distorted making urethroplasty with primary anastomosis or tissue substitution a difficult task [15, 16]. Further complicating the characteristics of these strictures is their location in the bulbomembranous urethra, higher incidence of postoperative urinary incontinence, erectile dysfunction, and fistula formation [16]. Therefore, these patients should be meticulously informed of the risks and benefits of pursuing surgical intervention.

The overall incidence of urethral stricture disease is also dependent on radiation dosage and the type of radiation used [9, 12]. Merrick et al. found the magnitude and extent of high dose radiation, mean membranous urethral dose, dose 20 mm proximal to the prostatic apex, and the duration of hormonal manipulation to be predictive of stricture formation after radiation therapy [17]. Compared with other side effects of radiation, stricture/stenosis is a relatively uncommon occurrence but is difficult to treat effectively.

## 4. Diagnosis and Evaluation

Patients presenting with LUTS following pelvic radiotherapy should undergo a thorough history and physical examination with special attention to the patency of the urethral meatus, suprapubic exam, and digital rectal examination. Furthermore, inquiries should be made regarding the dose and type of therapy the patient has received. When indicated, postvoid residual by ultrasound can assess bladder emptying [8, 17].

**Figure 1:**
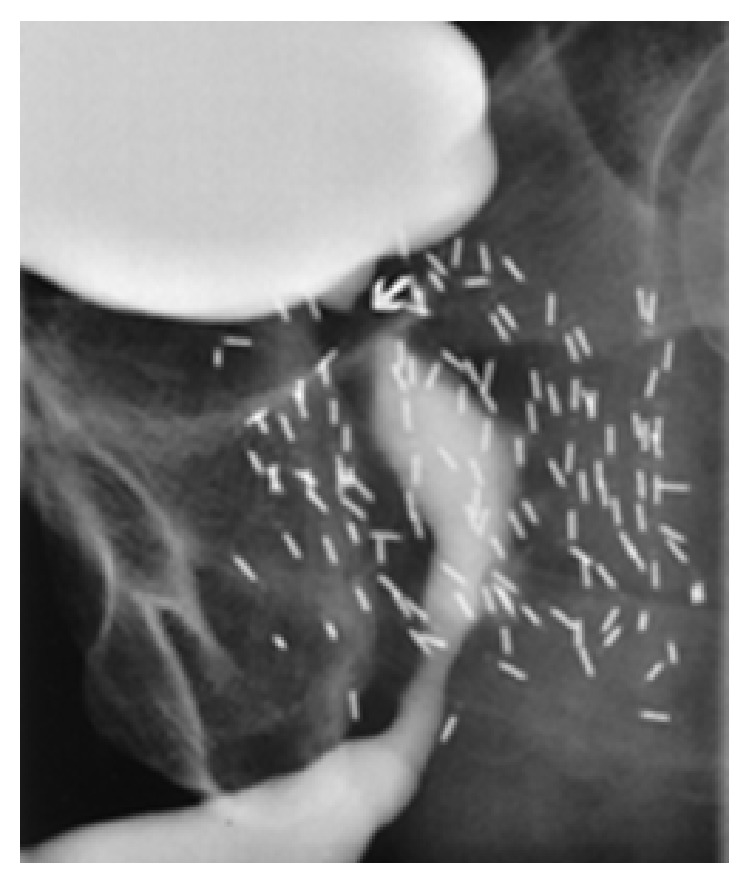
Bulbar and bladder neck stricture from combined EBRT and brachytherapy.* Credit to R. Santucci*.

Cystourethroscopy and retrograde urethrogram provide further detail on the location and length of the urethral stricture [12, 17]. However, exact delineation of the anatomy may be difficult due to distortion from the previously administered therapy. Assessment of external sphincter involvement and the length of the strictured segment are essential [8]. Retrograde urethrography offers the ability to determine the length and location of the obstruction (Figure 1).

If retrograde urethrogram is inconclusive, voiding cystourethrogram allows for full evaluation of the posterior urethra as well as the urethra proximal to the stricture. If a suprapubic tube is present, simultaneous antegrade endoscopy and retrograde urethrography can be performed [12].

Because of the potential deleterious effects of radiation therapy on the bladder, urodynamics can be helpful in evaluating bladder capacity prior to any potential surgery [12, 17]. For patients with bladder volumes less than 200 mL or severe detrusor instability, conservative measures to increase bladder volume may be attempted before reconstruction. However, other options such as bladder augmentation before reconstruction or urinary diversion can be discussed with the patient [12].

## 5. Treatment

Radiation induces an obliterative endarteritis that results in ischemia and fibrosis of the irradiated tissue. In the perioperative setting, these changes lead to compromised wound healing, altered tissue planes, and impaired blood supply of irradiated tissue [18]. Indeed these pathophysiologic changes induced by radiation are the underlying reasons why treatment can present a challenge.

Typical urethral stenosis after single-modality radiation treatment begins at the proximal bulbar urethra and extends through the membranous urethra and prostatic apex [2]. According to the experience of Mundy and Andrich, strictures after EBRT have an average stricture length of approximately 2 cm. Moreover, they report strictures secondary to combination of BT and EBRT are typically longer with nearly half being obliterative [8]. Short strictures are rare and when they happen, anastomotic repairs are rarely successful. Compared with strictures in BT patients, EBRT strictures are not commonly obliterative; they are less complicated to treat and therefore are theoretically amenable to anastomotic urethroplasty. Alternatively, tissue transfer repairs, such as grafts and/or flaps, are more likely to be appropriate and successful in those nonobliterative strictures which are not controlled by interval urethral dilatation [8, 19].

The gold standard for treatment of urethral strictures is urethroplasty with primary anastomosis or substitution urethroplasty being effective techniques depending on the stricture length [19]. Substitution urethroplasty can be accomplished with a graft and/or flap. The difference between the two methods is contingent on the presence (or lack thereof) of the grafted tissue's native blood supply. A graft is tissue that is moved from a donor site to a recipient site without its native blood supply. In contrast, flap tissues maintain their native blood supply on a pedicle that is transferred onto the recipient site [3, 19]. In the previously irradiated patient, several studies have shown urethroplasty to be efficacious. In general, direct-vision internal urethrotomy (DVIU) and dilation carry much higher failure rates than urethroplasty. Urethral stents have been studied in the setting of prostate cancer related urethral stricture disease and their application is discussed below though their use has fallen out of favor.

## 6. Urethral Dilation and Direct-Vision Internal Urethrotomy

The increased rate of complications associated with reconstruction of the radiated urethra underlies the initial selection of endoscopic therapy for the management of RT induced strictures, regardless of radiation modality. Endoscopic treatment of radiation-induced posterior urethral stenosis [PUS] has been associated with recurrences of approximately 40–60% regardless of the location or etiology [20]. In a study of 76 patients, Santucci and Eisenberg reported a stricture-free rate after first DVIU of 8% with median time to recurrence of 7 months. Subsequent urethrotomies were associated with decreased success rates with 0% stricture-free rate after the fourth and fifth procedures. As such, dilation and DVIU are advocated as temporizing measures, reserved for a select group of patients who have been counseled on the high likelihood of stricture recurrence until definitive curative reconstruction can be planned [1, 21].

Sullivan et al. assessed the nature and outcomes of urethral stricture disease in 38 patients who received high dose rate BT administered either as a boost to EBRT or as monotherapy. 92.1% of these patients experienced a stricture located in the bulbomembranous urethra with a mean time to diagnosis of 22 months. All strictures were initially managed with either dilation (*n* = 15) or DVIU (*n* = 20) with second-line therapy being performed in 17 cases (49%) via repeat dilation, DVIU, or intermittent self-catheterization. Only three cases (9%) required third-line therapy with one patient undergoing urethroplasty. While only one patient underwent invasive surgery with urethroplasty, nearly half of those who initially experienced a urethral stricture subsequently had second-line therapy to treat their stricture disease. However, as the study only provided a median follow-up time after treatment of the initial stricture of 16 months, long-term outcomes of treatment of BT-induced stricture disease cannot truly be reliably assessed based on this data alone [22].

Recently, Hudak et al. investigated the utility and counterproductive effects of repeat DVIU by reviewing 340 consecutive urethroplasties performed by a single surgeon to assess the association of repeat transurethral treatment with stricture complexity. Of 101 urethroplasties meeting inclusion criteria, it was discovered that repeat transurethral manipulation was associated with an eightfold increase in disease duration from stricture diagnosis to curative urethroplasty between patients who had undergone 0 to 1 prior DVIUs versus 2 or more (*p* < 0.001). Moreover, those who had undergone 2 or more previous DVIUs had significantly longer strictures (*p* = 0.001) and were more likely to undergo substitution urethroplasty. Furthermore, though not statistically significant, failure was more common in these patients versus those with 0 or 1 DVIU (12% versus 2%, *p* = 0.11) [23].

Intralesional injection of mitomycin C (MMC) has been assessed as an adjunct to DVIU owing to its ability to mitigate scar formation via inhibition of fibroblast proliferation. Farrell et al. reported a case series prospectively evaluating their experience with DVIU with intralesional MMC and short-term (1 month) clean intermittent catheterization (CIC). 37 patients were enrolled in the study and subsequently underwent DVIU with MMC and once daily CIC for treatment of refractory urethral stricture disease or bladder neck contracture. Radiation-induced urethral strictures were identified in 11 patients with 9 patients (81.8%) having received BT and the other 2 patients receiving EBRT and BT. Though no difference in stricture length was noted between patients with radiation-induced strictures and those without (mean 2.0 cm, *p* = 0.651), patients with prior radiation were noted to have deeper spongiofibrosis. Postoperatively, those with radiation-induced strictures did not experience a significant improvement in flow rate (*p* = 0.158) or PVR (*p* = 0.813) while those with strictures not related to radiation did experience significant improvements in these categories. The overall success rate was found to be 75.7% over the median follow-up period of 23 months. Recurrence-free success was 54.5% in the radiation cohort with a mean time to recurrence of 8 months [24]. The success rate of DVIU with MMC and CIC in patients with radiation-induced strictures is poorer compared to published data assessing urethroplasty [2, 18, 25–30] in this population and is within the estimated overall 40–60% success rate of DVIU/urethral dilation without MMC [20]. Therefore, while conceptually interesting, further large-cohort studies are necessary to the safety and efficacy of intralesional MMC in those with recalcitrant radiation-induced stricture disease.

## 7. Urethral Stents

The use of urethral stents have been described in the management of urethral stricture disease secondary to prostate cancer therapy. Eisenberg et al. described their experience with urethral stents for treatment of urethral stricture disease in 13 patients, of which 11 had previous history of prostate cancer therapy. The primary indication for urethral stenting versus reconstruction in these patients was to avoid the morbidity of surgery. Of these 11 patients, 3 received EBRT adjuvantly after radical prostatectomy and 4 received combined EBRT and BT with 2 also having undergone concomitant TURP. Overall, 6 of the 13 patients who underwent a urethral stent required additional procedures for stricture recurrence including 5 in previously irradiated patients. Furthermore, 8 of the 13 patients were subsequently rendered incontinent and willing patients underwent AUS placement [31].

In a subsequent study from the same institution, Erickson et al. assessed the efficacy of Urolume stents in 38 men with posterior urethral strictures secondary to prostate cancer treatment. 24 men (63%) received radiation therapy as either the primary treatment (16) or adjuvantly after radical prostatectomy (8). The modalities undertaken to administer radiation treatment were adjuvant EBRT in 8 patients, EBRT with salvage prostatectomy in 2 patients, BT in 8 patients, and BT with EBRT in 6 patients. After a mean follow-up of 2.3 ± 2.5 years, the authors reported an initial success rate of 47% improving to a final success rate of 89% after a total of 33 secondary procedures (including stent placement) in 19 men. Moreover, men who had received radiation therapy experienced recurrence sooner and required more secondary procedures. However, multivariate analysis failed to implicate radiation therapy as an independent risk factor for failure. The overall postoperative incontinence rate was found to be 82% with a higher rate in men who did versus did not receive previous radiation therapy (96% versus 50%, *p* < 0.001) [32].

Urolume stents are no longer commercially available in the United States and have globally fallen out of favor. However, the aforementioned studies advocate that urethral stenting is a reasonable treatment option for radiation-induced urethral strictures particularly when considering the significant postoperative morbidity patients may experience secondary to open excision. While initial success rates were dismal, secondary procedures led to vast improvements in urethral patency though many required yet further procedures to manage continence. Though incontinence and need for secondary procedures is expected, urethral stenting is a reasonable option for men unwilling or unable to undergo open urethral reconstruction.

## 8. Urethroplasty

Though urethroplasty is the most invasive approach to the treatment of urethral stricture disease, numerous studies have supported its use given the high rates of success. According to a review by Meeks et al., substitution urethroplasty using a buccal mucosal graft (BMG) has become the primary surgical treatment for long segment bulbar urethral strictures that are not suitable for anastomotic urethroplasty. The success rate for urethroplasty with BMG is between 81% and 96% with an estimated overall 15.6% failure rate for substitution urethroplasty [7]. Furthermore, as previously discussed, repeat DVIUs and/or dilation are destined to fail and may in fact reduce the efficacy of subsequent definitive therapy. In a review of 443 patients who underwent urethroplasty, Erickson et al. determined that a previous history of DVIU (*p* = 0.04) or urethroplasty (*p* = 0.03) was a significant factor predictive of urethroplasty failure [32]. Therefore, it stands to reason that further DVIUs and/or dilation should be avoided in favor of more definitive therapy.

Elliott et al. established this notion in 2006 when they prospectively assessed their management of 48 patients presenting with urethral stenosis or rectourinary fistula secondary to prostate cancer therapy. Of the 32 cases of stenosis, 14 occurred secondary to primary radiation therapy while 7 cases involved radical prostatectomy plus EBRT. 23 of 32 patients (73%) experienced successful repair of urethral stenosis, which involved anastomotic urethroplasty (19), flap urethroplasty (2), perineal urethrostomy (2), and urethral stent (9). Regardless of the location of the stricture (i.e., anterior versus posterior), success rates were nearly equal at 70% versus 73%. Moreover, the authors highlight prior EBRT as being a risk factor for urethral reconstruction failure as 9% of RP treated versus 50% of RP plus EBRT treated patients experienced failure after posterior urethroplasty. Of note, the authors excluded patients that had previous treatment with dilation, DVIU, or TUR and also did not subsequently manage any of the patients enrolled in the study with these treatment strategies. However, the study demonstrates that urethroplasty and urethral stenting are viable treatment options with acceptable rates of failure for patients presenting with radiation-induced stricture disease [25].

To further assess the efficacy urethroplasty for treatment of radiation-induced strictures, Meeks et al. performed a review of 30 men undergoing urethroplasty at three separate institutions. EBRT for prostate cancer was etiology of stricture disease in 15 men (50%) with brachytherapy in 7 and a combination of the two in 8. All strictures were noted to be in the proximal bulbar or membranous urethra and on average were 2.9 cm in length. At a mean follow-up of 21 months, 22 men (73%) experienced successful urethral reconstruction with the majority of individuals undergoing excision with primary anastomosis (80%). Incontinence was transient in 10% and persistent in 40%, with 13% subsequently undergoing placement of an artificial urinary sphincter [26].

Glass et al. retrospectively reviewed 29 men with urethral stricture following radiation treatment of prostate cancer of which 11 (38%) were treated with EBRT alone, seven (24%) had radical prostatectomy followed by adjuvant EBRT, seven (24%) had combined EBRT and brachytherapy, and four (14%) were treated with brachytherapy alone. The average stricture length was 2.6 cm. 22 of the cases were reconstructed with excision and primary anastomosis (EPA) (76%), substitution urethroplasty with buccal mucosa in five (17%), and fasciocutaneous flap onlay in two (7%). The overall success rate was 90% at a median follow-up of 40 months (range 12–83 months) with time to stricture recurrence ranging from 6 to 16 months. New onset of urge urinary incontinence was reported in two patients (7%) with one patient opting for an artificial urinary sphincter. Of note, one-third of the patients in this cohort underwent either DVIU or dilation, both of which have been previously shown to contribute to subsequent failure of urethroplasties [18, 33]. Therefore, despite previous treatment, urethroplasty was found to be highly successful in this series.

Further support for the efficacy of excision and primary anastomosis (EPA) for radiation-induced strictures was provided by a 2014 retrospective study conducted by Hofer and colleagues. Of the 72 men identified with radiation-induced urethral strictures, 66 (91.7%) underwent urethral reconstruction with EPA and the remaining 6 (8.3%) were treated with substitution urethroplasty using a graft or flap. Mean stricture length, which was determined intraoperatively, was 2.4 cm. Furthermore, stricture length was 2 cm or less in 37 of 65 men (56.1%) and greater than 2 cm in 28 men (42.4%). Stricture lengths were greater in those who underwent substitution urethroplasty (mean length 4.25 cm, range 3 to 7 cm). 46 (69.7%) men ultimately experienced successful reconstruction. Mean time to recurrence was found to be 10.2 months and was associated with stricture length greater than 2 cm (*p* = 0.013). Moreover, 12 (18.5%) men experienced new onset incontinence while the rate of ED remained stable. Radiotherapy type did not affect stricture length (*p* = 0.41), recurrence risk (*p* = 0.91), postoperative incontinence (*p* = 0.88), or erectile dysfunction (*p* = 0.53). Overall, EPA was found to be a successful treatment strategy for patients with radiation-induced strictures of the bulbomembranous urethra. Furthermore, the study indicates that men should be counseled on the development of de novo incontinence and the possible need for secondary procedures to provide adequate management [27].

In a 2015 study, Rourke et al. retrospectively reviewed outcomes in 35 patients undergoing urethroplasty for radiation-induced bulbomembranous stenosis. Of the 35 patients, 20 and 15 had stenosis related to EBRT and BT, respectively, with a mean stricture length of 3.5 cm. Nearly half of the patients enrolled in the study presented preoperatively with an indwelling suprapubic catheter indicating baseline. Reconstruction was performed using anastomotic urethroplasty in 23 patients (65.7%) with 12 patients requiring tissue transfer via buccal mucosa graft (20.0%) or penile island flap (14.3%). With 50.5 months of follow-up, thirty patients (85.7%) achieved cystoscopic patency with no significant difference between techniques (*p* = 0.32). 31.4% of patients experienced a reportable 90-day complication all of which were Clavien Grades I-II [2].

The work of Rourke et al. indicates that urethroplasty is efficacious in radiation-induced urethral stenosis. However, even in well-selected patients (i.e., those without extensive prostatic necrosis, cavitation, prostatosymphyseal fistula, osteomyelitis, or small functional bladder capacity) minor complications were fairly common albeit acceptable and manageable. Despite achieving urethral patency many patients continued to experience bothersome LUTS as well as ED and incontinence. This suggests that even though commendable urethral patency rates may be attained, urethroplasty cannot alone mitigate and may even exacerbate, many of the concomitant complaints experienced by this patient population [2].

While the aforementioned studies have largely assessed the efficacy of anastomotic urethroplasties, long-term outcomes reported in men undergoing substitution urethroplasty have demonstrated higher recurrence rates than anastomotic techniques. The failure of substitution urethroplasties is further exacerbated by the use of donor graft or flap tissue that has been irradiated, which can compromise the effects of the previous radiation exposure.

An abstract published by Kuhl et al. specifically assessed the outcomes of buccal mucosa graft (BMG) urethroplasties for the treatment of radiation-induced urethral strictures. Of the 20 patients enrolled in the study with available data, 75% of treated strictures were within the bulbomembranous urethra and less than 6 cm in length. The success rate was found to be 60% after 25 months of follow-up. Postoperatively, patients experienced an improvement in flow rate from 8.4 to 25.6 mL/s, which trended towards significance (*p* < 0.07) in flow rate. Furthermore, 29% and 10% of the patients with preoperative incontinence experienced worsening or de novo incontinence, respectively. However, despite these postoperative changes in continence, BMG substitution urethroplasties were deemed to be successful with high rates of patient satisfaction [28].

Long segment strictures have been found to be challenging to treat. When treated with traditional dorsal or ventral onlay approaches, these strictures carry a high risk of recurrence due to the lack of a well-vascularized graft bed. Moreover, previously irradiated fields are often poorly vascularized thereby impeding wound healing [12, 29, 30]. In order to promote neovascularity and healing of these reconstructions, Palmer et al. assessed the use of a gracilis muscle flap to provide a well-vascularized graft bed for buccal graft substitutions. After performing a ventral buccal graft onlay, the authors describe harvesting and rotating gracilis muscle onto the perineum and buttressing the muscle to the graft. 20 patients with long segment urethral strictures secondary to various etiologies including radiation therapy in 45% (9 of 20) were retrospectively reviewed. Before surgery, 18 patients (90%) had undergone dilatation and/or endoscopic incision. Strictures were located in the posterior urethra with or without bulbar urethral involvement in 50% of cases (10), the bulbomembranous urethra in 35% (7), the bulbar urethra in 10% (2), and the proximal pendulous urethra in 5% (1) with a mean stricture length of 8.2 cm. Urethral reconstruction was found to be successful in 16 cases (80%) at a mean follow-up of 40 months. Mean time to recurrence was observed to be 10 months with 5 patients (25%) experiencing postoperative incontinence requiring an artificial urinary sphincter. Despite significant preoperative risk factors, the authors demonstrate the efficaciousness of substitution urethroplasty with gracilis flap thereby supporting its use in complex patients with a previous history of radiation therapy. However, despite the encouraging results, the study is limited by its retrospective nature and small sample size [30].

Ahyai et al. recently published their experience with ventral onlay buccal mucosa graft urethroplasties in patients with radiation-induced strictures. 35 of the 38 men (92.1%) included in the study underwent radiotherapy for treatment of prostate cancer with 64.9% exclusively undergoing EBRT. BT was performed in 8 patients (21.6%) with EBRT and BT being performed in combination in 6 patients (13.5%). The median length of strictures treated was 3.0 cm. The mean length of implanted buccal graft was 4.9 cm. 27 patients had undergone previous urethral dilation or DVIU. After a median follow-up of 26.5 months, the overall success rate was 71.1% with 4 patients (10.5%) experiencing de novo incontinence and 11 patients (28.9%) experiencing recurrence. Though limited by its retrospective design and small sample size, the study indicates that ventral onlay buccal mucosa urethroplasty is an acceptable treatment strategy with results similar to EPAs particularly for patients with strictures greater than 1 cm [34].

## 9. Conclusions

Men with urethral strictures secondary to nonsurgical forms of treatment for prostate cancer represent a challenging cohort to treat. Published data suggests that radiation-induced strictures are best treated with urethroplasty via anastomic or substitution techniques. Patients should be counseled on the high likelihood of stricture recurrence after DVIU or dilation. Moreover, a detailed discussion should take place regarding development or worsening of incontinence after treatment with urethroplasty. Further studies are required to assess the nature and treatment of cryotherapy and HIFU-induced strictures.
